# Supplementary material for: Epigenomic mapping identifies an enhancer repertoire that regulates cell identity in bladder cancer through distinct transcription factor networks
Source: Oncogene. 2023 Mar 22;42(19):1524–42. doi: 10.1038/s41388-023-02662-1 (PMC10162941; doi:10.1038/s41388-023-02662-1)
Supplement: Supplementary file 1 — Supplemental Information [file 41388_2023_2662_MOESM1_ESM.docx]

**Epigenomic mapping identifies an enhancer repertoire that regulates cell identity in bladder cancer through distinct transcription factor networks: Supplemental information**

[Additional material and methods: 2](#_Toc114240706)

[Antibodies 2](#_Toc114240707)

[siRNA 2](#_Toc114240708)

[CRispR vectors and guides 2](#_Toc114240709)

[Tools, Web sites, Data Base 2](#_Toc114240710)

[Commercial Assays 3](#_Toc114240711)

[Supplemental tables titles 4](#_Toc114240712)

[Table S1: 4](#_Toc114240713)

[Table S2: 4](#_Toc114240714)

[Table S3: 4](#_Toc114240715)

[Table S4 4](#_Toc114240716)

[Table S5: 4](#_Toc114240717)

[Table S6: 4](#_Toc114240718)

[Table S7: 4](#_Toc114240719)

[Supplemental figures titles 4](#_Toc114240720)

[Figure S1: Epigenetic landscape of BLCA. 4](#_Toc114240721)

[Figure S2: Chromatin State Map of BLCA 4](#_Toc114240722)

[Figure S3: Identification of the bladder super-enhancer repertoire and subtype specificities 4](#_Toc114240723)

[Figure S4: Master Regulators 4](#_Toc114240724)

[Figure S5: FOXA1 binding profile 4](#_Toc114240725)

[Figure S6: FOXA1 regulates inflammation and cellular identity 4](#_Toc114240726)

[Figure S7: ZBED2, a novel Basal-associated TF involved in inflammation dampening 4](#_Toc114240727)

# Additional material and methods:

## Antibodies

| Antibodies | Brand | Reference |
| --- | --- | --- |
| Histone H3K27acetyl | Abcam | ab4729 |
| Histone H3K27me3 | Active Motif | 39155 |
| Histone H3K9me3 | Active Motif | 39161 |
| H3K4me3 | Abcam | ab8580 |
| FOXA1 | Abcam | ab23738 |
| CTCF | Millipore | 07-729 |
| GATA3 (IHC) | Diagomics | L50-823 |
| CK5/6 (IHC) | Diagomics | EP24/EP67 |
| P16(IHC) | Diagomics | IHC116 |
| Mouse HRP (secondary, IHC) | Vector | MP-7402 |
| Rabbit Ap secondary Ab (IHC) | Enzo | ENZ-ACC110-0150 |

## siRNA

| Target gene | Brand | Reference |
| --- | --- | --- |
| siFOXA1_1 | Ambion | s6688 |
| siFOXA1_2 | Ambion | s6689 |
| siControl_1 | Ambion | 4390843 |
| siControl_2 | Ambion | 4390846 |
| siZBED2_1 | Ambion | s35780 |
| siZBED2_2 | Ambion | s35781 |
| siFOXA1 pool | Dharmacon | L-010319-00 |
| siControl pool | Dharmacon | D-001810-10- 05 |

## CRispR vectors and guides

| Vector | Brand | Reference |  |
| --- | --- | --- | --- |
| Cas9 + Scafold gRNA | VectorBuilder | VB161201-1064vnm | GTTTTAGAGCTAGAAATAGCAAGTTAAAATAAGGCTAGTCCGTTATCAACTTGAAAAAGTGGCACCGAGTCGGTGC |
| Cas9 + FOXA1 gRNA (1) | VectorBuilder | VB161130-1033fmt | GCACTGCAATACTCGCCTTA |
| Cas9 + FOXA1 gRNA (2) | VectorBuilder | VB161130-1031bdf | CATGTTGCCGCTCGTAGTCA |

## **Tools, Web sites, Data Base**

| Tool | Source | Reference |
| --- | --- | --- |
| Viper R package v 1.22 | Bioconductor |  |
| ARACNe-AP | https://github.com/califano-lab/ARACNe-AP |  |
| R | https://www.r-project.org/ | 4.1.0 |
| Rstudio | https://www.rstudio.com/ | 1.4.1106 |
| Diffbind | Bioconductor | 2.16.0 |
| DESeq2 | Bioconductor | 1.32.0 |
| fgsea | Bioconductor | 1.18.0 |
| clusterprofiler | Bioconductor | 4.0.0 |
| ReactomePA | Bioconductor | 1.36.0 |
| pheatmap | CRAN | 1.0.12 |
| ComplexHeatmap | Bioconductor | 2.8.0 |
| ChIPseeker | Bioconductor | 1.28.3 |
| FactoMineR | CRAN | 2.4 |
| consensusMIBC | https://github.com/cit-bioinfo/consensusMIBC | 1.1.0 |
| igraph | CRAN | 1.2.6 |
| EnhanceVolcano | Bioconductor | 1.10.0 |
| ggpubr | CRAN | 0.4.0 |
| GSVA | Bioconductor | 1.40.1 |
| TCGAbiolinks | Bioconductor | 2.20.0 |
| msigdbr | CRAN | 7.4.1 |
| classifyNMIBC | https://github.com/sialindskrog/classifyNMIBC |  |
| batchelor | Bioconductor |  |
| Easeq | https://easeq.net/ |  |
| Homer | http://homer.ucsd.edu/homer/ | v4.11 |
| Cistrome | http://dbtoolkit.cistrome.org/ |  |
| Washu epigenome browser | http://epigenomegateway.wustl.edu/legacy/ | legacy |
| ChromHMM | http://compbio.mit.edu/ChromHMM/ | v1.23 |
| GraphPad Prism |  | v8.4.3 |
| GREAT | http://great.stanford.edu/public/html/ |  |
| Bedtools | https://bedtools.readthedocs.io/en/latest/ |  |
| Bowtie 1.0.0 |  | 1.0.0 |
| MACS2 |  | v2.1.0.20140616 |
| ROSE | (Lovén et al., 2013; Whyte et al., 2013) |  |

## **Commercial Assays**

| Assay | Brand | Reference |
| --- | --- | --- |
| iDeal ChIP-seq Kit for Histones | Diagenode |  |
| ChIP-IT High Sensitivity kit | Active Motif |  |
| NEXTflex ChIP-Seq Kit | Bioo Scientific | 5143-02 |
| NEXTflex ChIP-Seq Barcodes | Bioo Scientific | #514120 |
| Diagenode MicroPlex Library Preparation kit v2 | Diagenode |  |
| Bioanalyzer system 2100 |  |  |
| HiSeq 4000 platform | Illumina |  |
| Hi-Seq 2500 | Illumina |  |
| Novaseq 6000 | Illumina |  |
| TruSeq Stranded mRNA Library preparation kit | Illumina |  |
| Nugen kit |  |  |
| KAPA library quantification kit | Roche |  |
| QuantSeq FWD 3’mRNA Seq LEXOGEN Standard | CliniSciences |  |
| Agencourt AMPure XP beads | Beckman | #A63881 |
| Lipofectamine RNAi max | Invitrogen | 13778150 |
| Fugene HD | Promega | E2311 |
| CellTiter-Glo® Luminescent Cell Viability Assay | Promega | G7570 |
| RNeasy kit | Qiagen |  |
| RT high Capacity Kit | Applied | 4368814 |
| SYBR Green | Roche | 04707516001 |
| Probe Master | Roche | 04707494001 |
| EnVision FLEX, High pH | Agilent | GV800 |
| KIT ImmPACT DAB | Vector | SK-4105 |
| Kit ImmPACT red substrate alkaline phosphatase |  |  |

# Supplemental tables titles

Table S1: Annotation table of samples indicating Tumour cell content, consensus classification and statistics. Tumor-reference based molecular subtyping for BCa cell lines.

Table S2: ROSE SE table for each sample

Table S3: Consensus SE and analysis

Table S4: Reactome pathway analysis of Ba/Sq vs Luminal SEs

Table S5: H3K27ac peaks differential analysis inside SE and motif enrichment analyses using Cistrome and Homer

Table S6: DEG in siFOXA1 vs Controls

Table S7: DEG in CRispR mutant FOXA1 vs Controls

# Supplemental figures titles

## **Figure S1:** Epigenetic landscape of BLCA.

## **Figure S2:** ChIPseq quality controls

## **Figure S3:** Chromatin State Map of BLCA

## **Figure S4:** Identification of the bladder enhancer repertoire and subtype specificities

## **Figure S5:** Master Regulators

## **Figure S6:** FOXA1 binding profile

## **Figure S7:** FOXA1 regulates inflammation and cellular identity

## **Figure S8:** ZBED2, a novel Basal-associated TF involved in inflammation dampening
