# Supplementary figures and images for: Epigenomic mapping identifies an enhancer repertoire that regulates cell identity in bladder cancer through distinct transcription factor networks
Source: Oncogene. 2023 Mar 22;42(19):1524–42. doi: 10.1038/s41388-023-02662-1 (PMC10162941; doi:10.1038/s41388-023-02662-1)

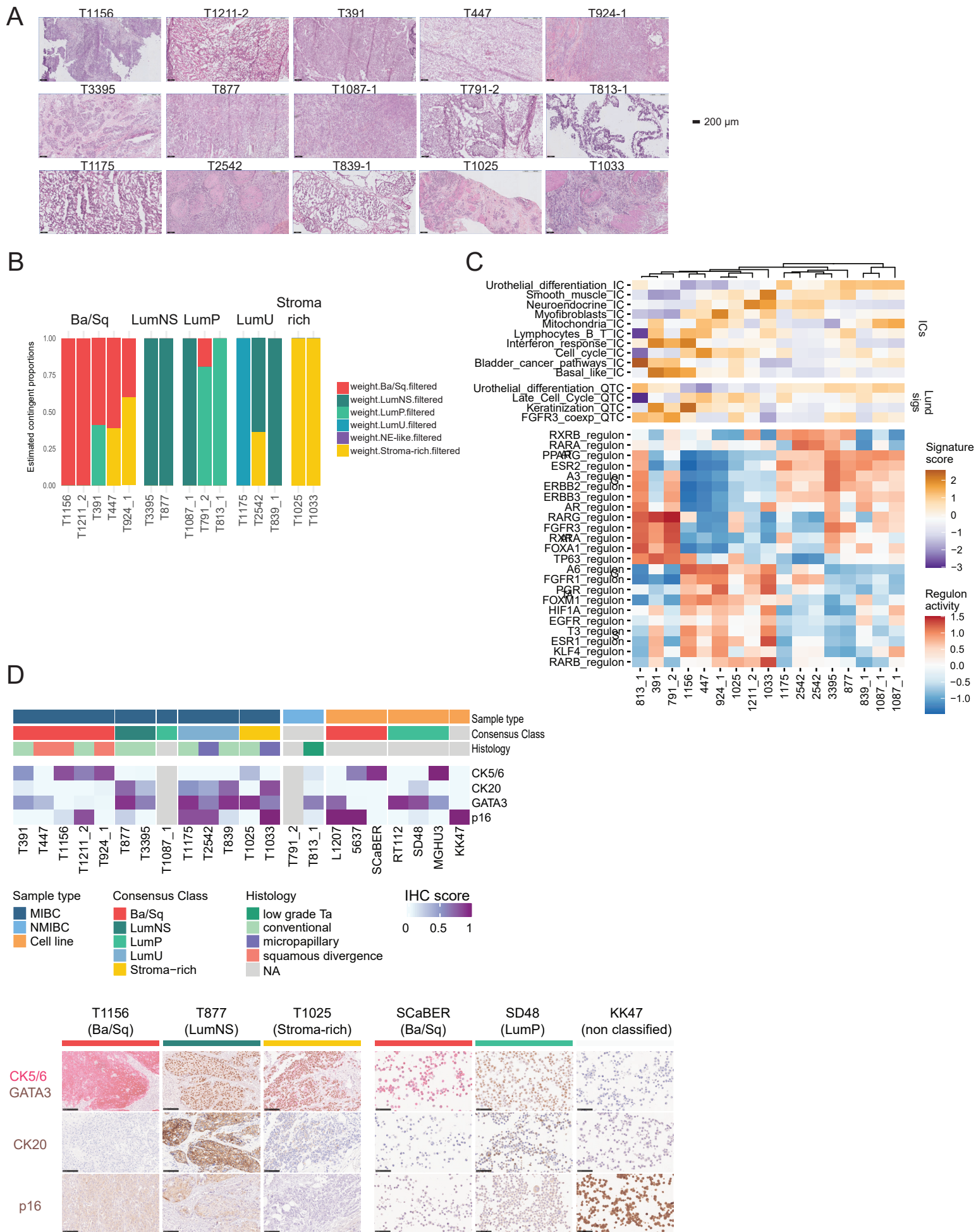

Supplement: Supplementary file 2 — Figure S1 [file 41388_2023_2662_MOESM2_ESM.pdf]
