# Supplementary material for: Epigenomic mapping identifies an enhancer repertoire that regulates cell identity in bladder cancer through distinct transcription factor networks
Source: Oncogene. 2023 Mar 22;42(19):1524–42. doi: 10.1038/s41388-023-02662-1 (PMC10162941; doi:10.1038/s41388-023-02662-1)
Supplement: Supplementary file 3 — Figure S2 [file 41388_2023_2662_MOESM3_ESM.pdf]

A

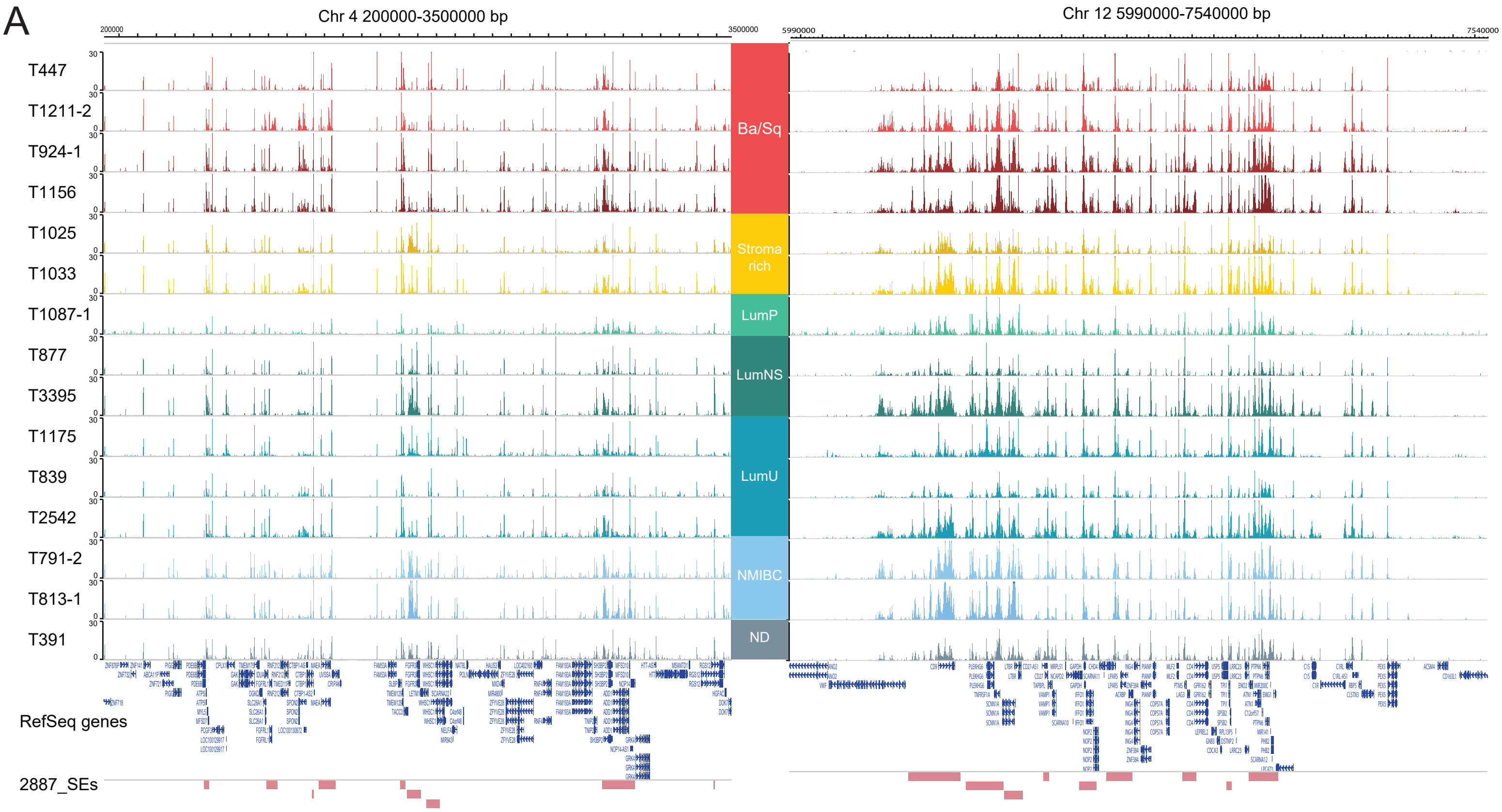

B

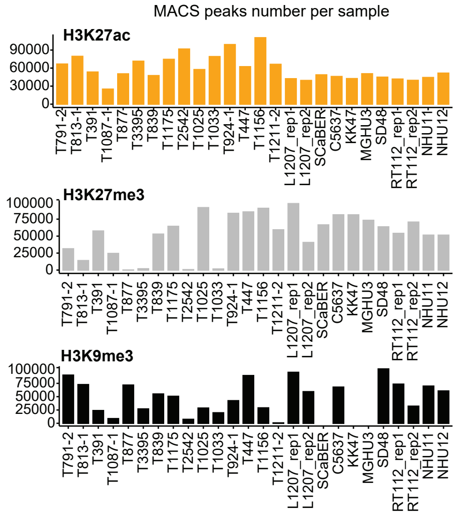

C

Sample	Mark	Number of peaks	total read	read in peaks	FRiP
T1025	H3K27ac	57480	12898446	6780203	**0.52566**
T1033	H3K27ac	79083	23252169	11227875	**0.482874**
T1087-1	H3K27ac	24964	9868799	2169127	**0.219796**
T1156	H3K27ac	110445	36402472	19359698	**0.531824**
T1175	H3K27ac	74464	28222155	11849954	**0.419881**
T1211-2	H3K27ac	66171	31066987	10117684	**0.325673**
T2542	H3K27ac	91628	26268361	10266973	**0.390849**
T3395	H3K27ac	71353	29813837	14364869	**0.481819**
T391	H3K27ac	57369	32259279	14840331	**0.460033**
T447	H3K27ac	62273	16511796	10594952	**0.64166**
T791-2	H3K27ac	66645	34200842	17422092	**0.509405**
T813-1	H3K27ac	79452	26769347	16614011	**0.620636**
T839	H3K27ac	47443	11899135	6135329	**0.515611**
T877	H3K27ac	50386	13253291	6569751	**0.495707**
T924-1	H3K27ac	99034	38174186	21101523	**0.552769**
T1025	H3K27me3	91945	17523244	6989698	**0.398882**
T1033	H3K27me3	1531	32907632	168192	**0.005111**
T1087-1	H3K27me3	24447	13475044	1492759	**0.11078**
T1156	H3K27me3	90948	23818238	6759895	**0.283812**
T1175	H3K27me3	64395	26637172	6755173	**0.253599**
T1211-2	H3K27me3	59279	32004727	6898519	**0.215547**
T2542	H3K27me3	566	28325631	172339	**0.006084**
T3395	H3K27me3	1863	25130663	222722	**0.008863**
T391	H3K27me3	57510	22666982	4802169	**0.211857**
T447	H3K27me3	85587	27760311	8445860	**0.304242**
T791-2	H3K27me3	31347	12101578	1567048	**0.129491**
T813-1	H3K27me3	13938	52702312	608480	**0.011546**
T839	H3K27me3	52912	13348050	2562277	**0.191959**
T877	H3K27me3	7	29592691	1016	**3.43E-05**
T924-1	H3K27me3	83485	26832708	6360990	**0.237061**
T1025	H3K9me3	28570	5239795	1250389	**0.238633**
T1033	H3K9me3	19704	37401410	1247093	**0.033343**
T1087-1	H3K9me3	8777	9827987	1104504	**0.112384**
T1156	H3K9me3	28957	20658261	1155253	**0.055922**
T1175	H3K9me3	50631	17941560	3001338	**0.167284**
T1211-2	H3K9me3	740	23536967	337744	**0.01435**
T2542	H3K9me3	7393	32151810	867307	**0.026975**
T3395	H3K9me3	26974	36299887	1695578	**0.04671**
T391	H3K9me3	23883	12205751	1353281	**0.110872**
T447	H3K9me3	89610	21470059	5073066	**0.236286**
T791-2	H3K9me3	90420	43887296	9374620	**0.213607**
T813-1	H3K9me3	72650	23474222	1681764	**0.071643**
T839	H3K9me3	55007	11698270	2449291	**0.209372**
T877	H3K9me3	71717	13963038	2888492	**0.206867**
T924-1	H3K9me3	42422	24931930	1518931	**0.060923**

Sample	Mark	Number of peaks	total read	read in peaks	FRiP
5637	H3K27ac	45940	48069709	20721568	**0.431073**
5637	H3K27me3	81381	38545596	16184847	**0.419888**
5637	H3K9me3	67899	38880258	7699164	**0.198022**
KK47	H3K27me3	81295	2927476	53790455	**0.054424**
KK47	H3K27ac	42479	2641660	49789214	**0.053057**
L1207	H3K27ac	49958	30656612	79371958	**0.38624**
L1207	H3K27me3	99012	28898848	94964172	**0.304313**
L1207	H3K9me3	120513	19348686	74700084	**0.259018**
MGHU3	H3K27me3	73221	1712698	46163480	**0.037101**
MGHU3	H3K27ac	50534	1644454	39826658	**0.04129**
NHU11	H3K9me3	69783	1113263	58946442	**0.018886**
NHU11	H3K27me3	51400	1480880	46449018	**0.031882**
NHU11	H3K27ac	44280	1875773	52280305	**0.035879**
NHU12	H3K9me3	60671	544925	57677279	**0.009448**
NHU12	H3K27me3	51384	1772139	71573711	**0.02476**
NHU12	H3K27ac	51542	1559343	43893510	**0.035526**
RT112	H3K27ac	46513	47340406	1.17E+08	**0.40384**
RT112	H3K27me3	118688	21180874	68708970	**0.308269**
RT112	H3K9me3	64029	9744421	70999979	**0.137245**
SCaBER	H3K27me3	66506	1673182	32874254	**0.050896**
SCaBER	H3K27ac	48691	2606122	53174952	**0.04901**
SD48	H3K27ac	44822	37894501	19850888	**0.523846**
SD48	H3K27me3	63627	76768376	16525476	**0.215264**
SD48	H3K9me3	102050	39382228	6221312	**0.157973**

Figure S2: ChIPseq Quality controls

A) Washu Epigenome browser view of H3K27ac ChIPseq in tumors (bigwig) at 2 large windows on chromosome 4 (around FGFR3 gene) and chromosome 12 (around GAPDH gene).
B) Macs2 Peaks number per sample for each tested histone marks. C) Fraction of Reads in Peaks statistics from MACS2 peaks.
