# Supplementary material for: Epigenomic mapping identifies an enhancer repertoire that regulates cell identity in bladder cancer through distinct transcription factor networks
Source: Oncogene. 2023 Mar 22;42(19):1524–42. doi: 10.1038/s41388-023-02662-1 (PMC10162941; doi:10.1038/s41388-023-02662-1)
Supplement: Supplementary file 4 — Figure S3 [file 41388_2023_2662_MOESM4_ESM.pdf]

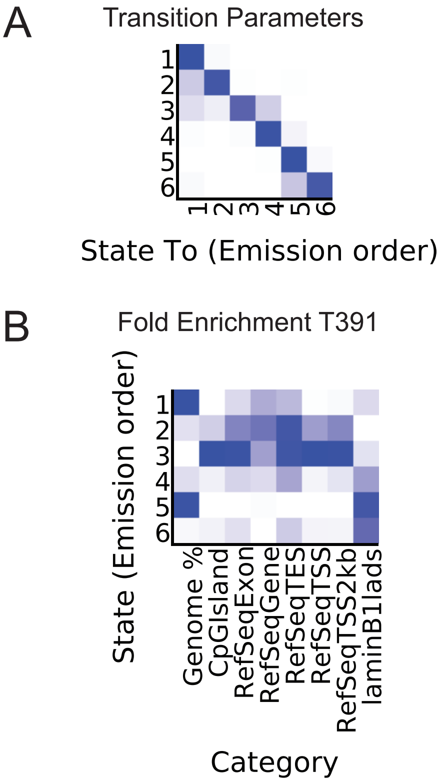

C

Number of genes by corresponding chromatin states at the TSS in the samples analyzed							
SampleID		Chromatin states					
		E1	E2	E3	E4	E5	E6
Tumors	T391	18208	15288	3957	5046	13327	1905
	T447	22317	13018	4554	4913	10191	2740
	T791	18281	16266	1508	3089	13998	4591
	T813	22347	17615	660	6878	7516	2713
	T839	17005	13413	4026	4468	16213	2607
	T877	19610	15749	1208	4921	13901	2338
	T924	20687	17859	2453	5972	8838	1919
	T1025	17521	12835	5371	6415	14324	1268
	T1033	22513	17411	1090	3812	10378	2525
	T1087	16023	11205	1545	4646	21957	2353
	T1156	20487	16521	3700	5278	9553	2191
	T1175	23555	17591	1932	5421	7391	1837
	T1211	25165	15595	1514	7841	6998	619
	T2542	24860	17683	1117	2825	9576	1673
	T3395	22156	17572	1248	2888	11655	2211
Cell lines	5637	17738	12808	886	10429	10975	4897
	KK47	21568	13365	798	10934	10457	611
	L1207	15053	14311	1210	12992	8377	5788
	MGHU3	24841	13559	336	8701	9577	719
	NHU121621	16746	13278	2040	9808	9349	6511
	NHU161130	16851	13031	2959	8665	9755	6469
	RT112	14143	12719	2382	12746	11876	3861
	Scabber	24777	14638	819	8398	8618	483
	SD48	15273	10536	2757	10649	13771	4742
TSS, transcription start site							

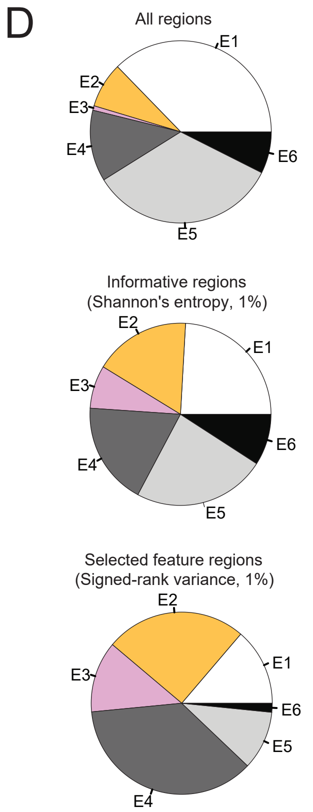

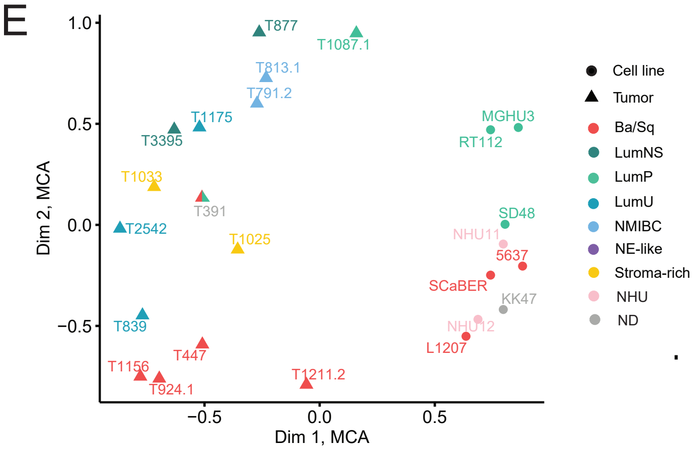

Figure S3: Chromatin State Map of BLCA
A) Transition parameters from CHromHMM state output.
B) ChromHMM output, example of Fold enrichment over genome categories (Tumour T391)
C) Number of genes by corresponding chromatin states at the TSS in the samples analyzed.
D) Pie chart illustrating chromatin state of all regions (entire genome), informative regions and top 1% varying regions by signed-rank.
E) MCA of Chromatin states on 1% most varying genome segments.
