# Supplementary material for: Epigenomic mapping identifies an enhancer repertoire that regulates cell identity in bladder cancer through distinct transcription factor networks
Source: Oncogene. 2023 Mar 22;42(19):1524–42. doi: 10.1038/s41388-023-02662-1 (PMC10162941; doi:10.1038/s41388-023-02662-1)
Supplement: Supplementary file 5 — Figure S4 [file 41388_2023_2662_MOESM5_ESM.pdf]

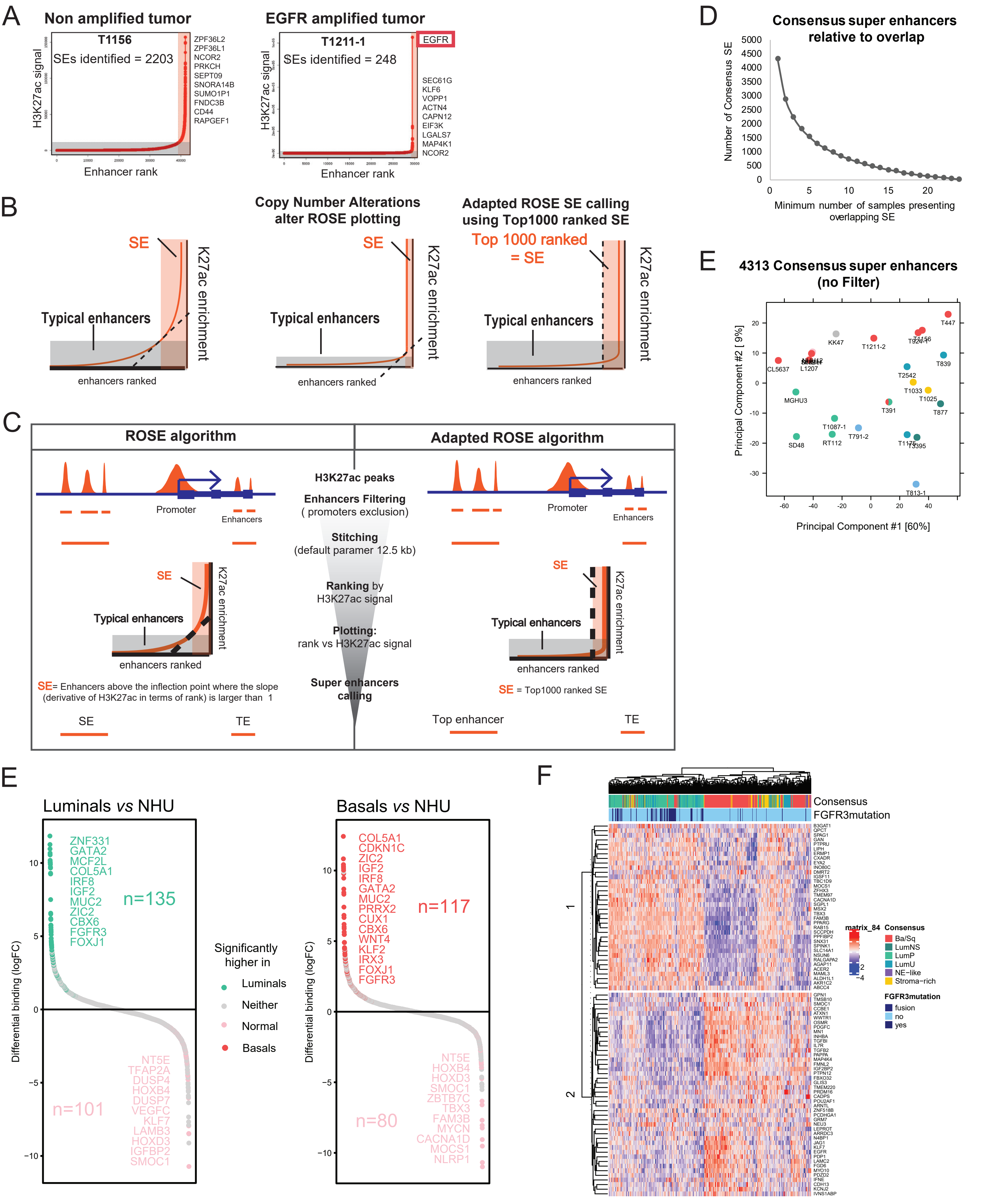

Figure S4: Identification of the bladder enhancer repertoire and subtype specificities

A) Examples of ROSE plotting in tumor T1156 without gene amplification, and in T1211-2 harboring a large EGFR amplification. Regions corresponding to the dots to the right of the curve's inflexion point are considered super-enhancers by the algorithm. In tumor T1211-2 with a gene amplification (red box), the number of identified SEs is lower. The symbols of the gene closest to the top 10 ranked regions are shown at the right of each plot. B) Schematic view of ROSE ranked enhancer plotting, the bias induced by Copy number alterations and how the pipeline was adapted to reduce variations in number of detected SE. C) Scheme comparing the default ROSE SE identification pipeline with the adapted pipeline used in our study. D) Graph showing the number of consensus SEs detected according to the overlap filter applied. E) PCA of H3K27ac signal inside ROSE unfiltered consensus SE set (n=4313) for all samples.E) Fold Change plots for Differential enhancers between Ba/Sq and LumP vs NHU (including NMIBC), Significance by pvalue <0.05. F) TCGA expression Heatmap of differential SEs assigned genes (FDR<0.05,Clustering using Complete method).
