# Supplementary material for: Epigenomic mapping identifies an enhancer repertoire that regulates cell identity in bladder cancer through distinct transcription factor networks
Source: Oncogene. 2023 Mar 22;42(19):1524–42. doi: 10.1038/s41388-023-02662-1 (PMC10162941; doi:10.1038/s41388-023-02662-1)
Supplement: Supplementary file 6 — Figure S5 [file 41388_2023_2662_MOESM6_ESM.pdf]

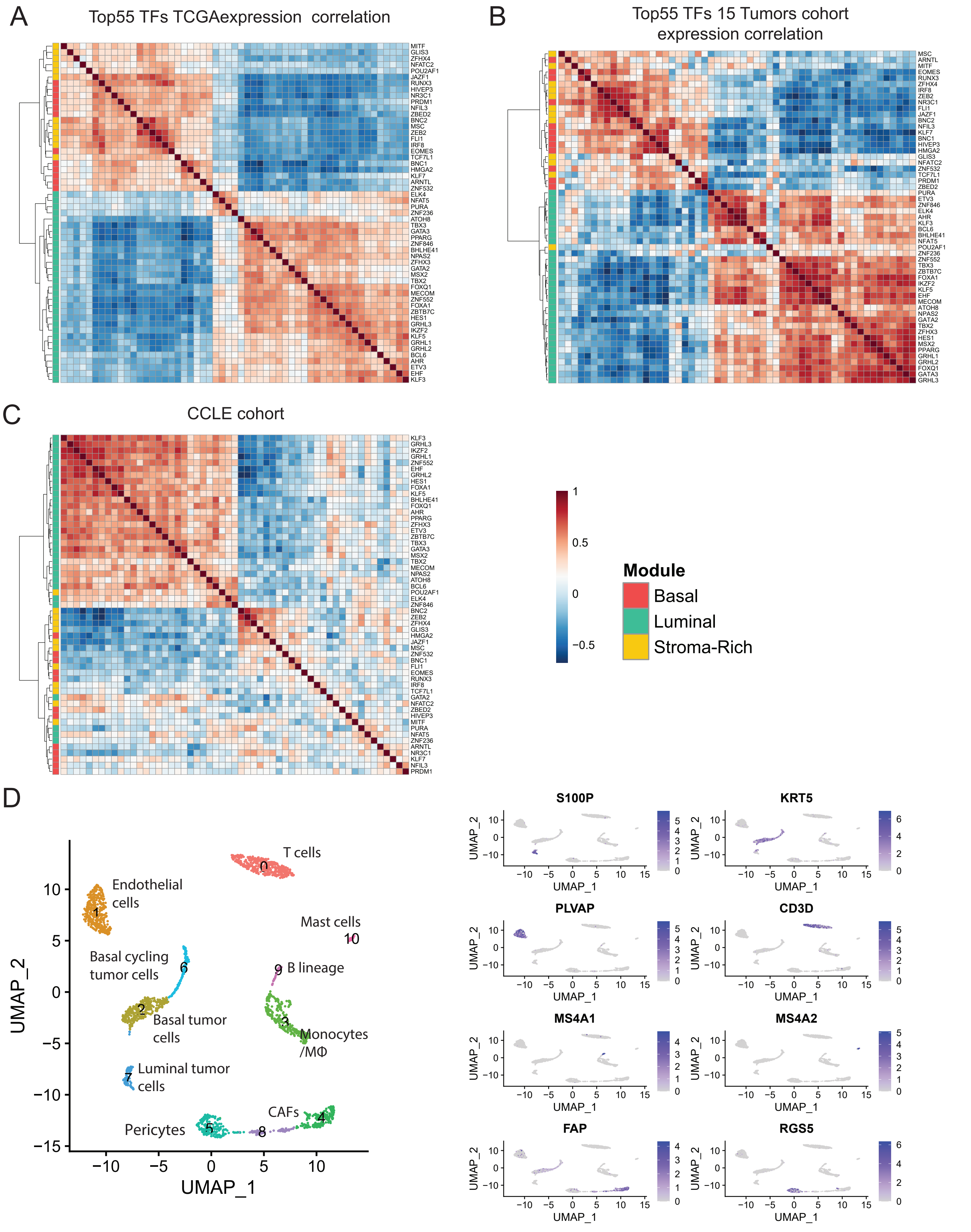

Figure S5: Master Regulators

A, B, C) Top55 TFs correlation of expression in TCGA (A), our 15 tumors cohort (B) and in CCLE (C). D) Single cell RNAseq analysis of one Bladder Cancer tumour with both Basal and Luminal Population (GSM4307111). Right panels, expression of key markers in each single cell cluster.
