# Supplementary material for: Epigenomic mapping identifies an enhancer repertoire that regulates cell identity in bladder cancer through distinct transcription factor networks
Source: Oncogene. 2023 Mar 22;42(19):1524–42. doi: 10.1038/s41388-023-02662-1 (PMC10162941; doi:10.1038/s41388-023-02662-1)
Supplement: Supplementary file 7 — Figure S6 [file 41388_2023_2662_MOESM7_ESM.pdf]

A

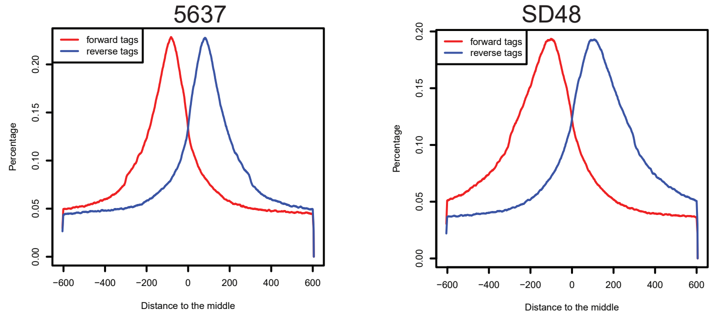

Sample	Mark	Number of peaks	total read	read in peaks	FRiP
5637	FOXA1	39445	60479137	6625091	**0.109543**
SD48	FOXA1	61083	36509534	16078870	**0.440402**
5637	CTCF	57615	47101873	10581044	**0.224642**
SD48	CTCF	59867	40490172	10851842	**0.268012**
5637	H3K27ac	45940	48069709	20721568	**0.431073**
SD48	H3K27ac	44822	37894501	19850888	**0.523846**
5637	H3K4me3	17321	54778008	48037860	**0.876955**
SD48	H3K4me3	19852	55411490	39518632	**0.713185**

B

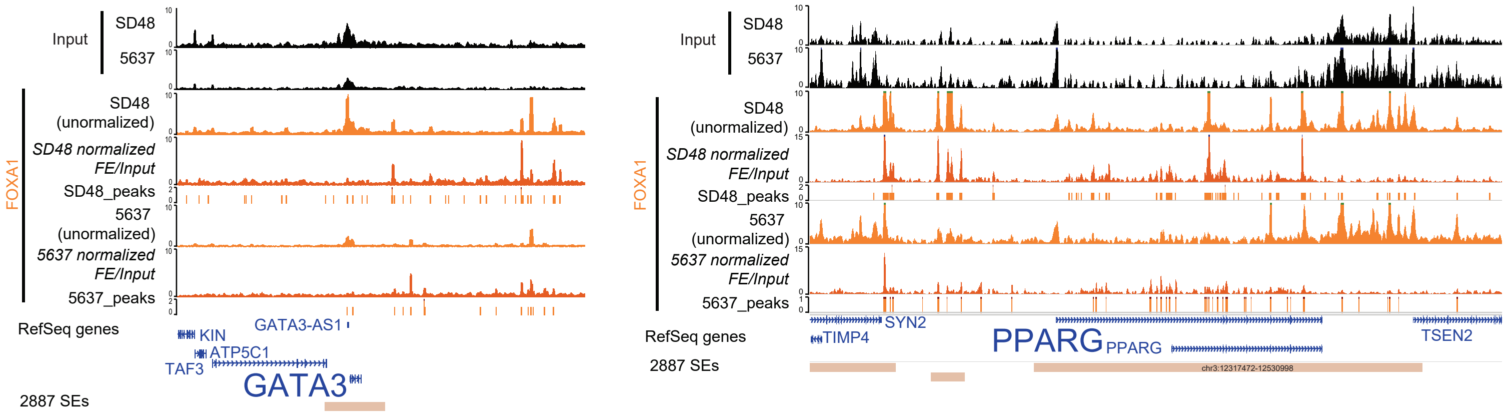

C

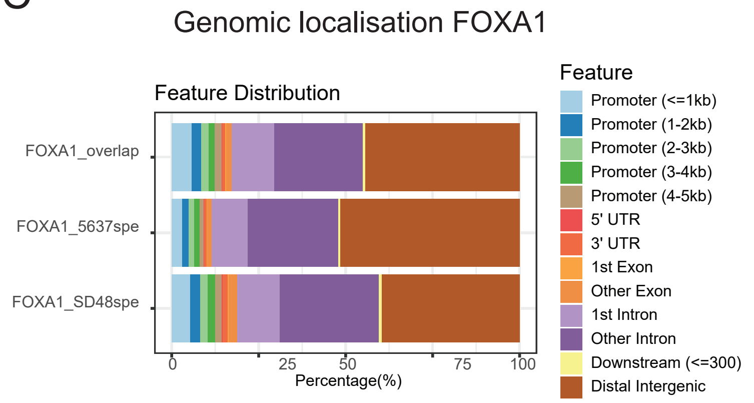

D

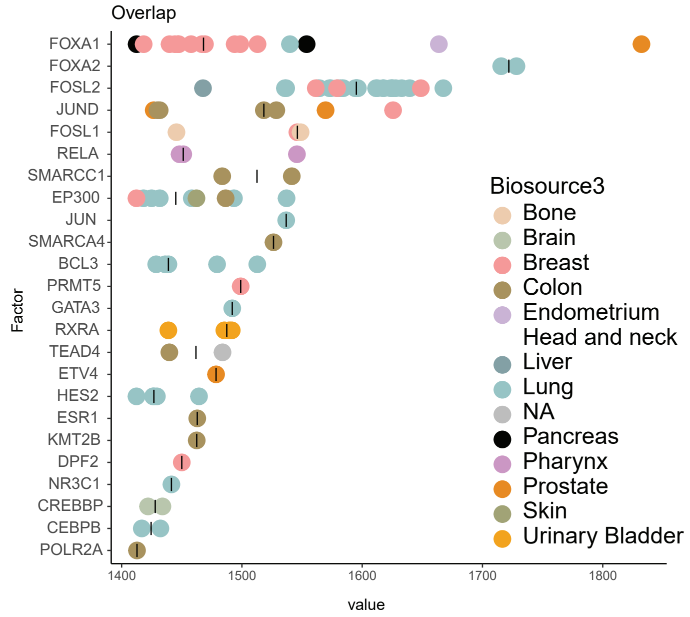

E

Homer motif enrichment FOXA1
SD48 specific peaks over 5637 specific peaks

Motif Name		Log P-value
Foxa2(Forkhead)	Liver-Foxa2-ChIP-Seq(GSE25694)	-3,38E+03
GATA3(Zf)	iTreg-Gata3-ChIP-Seq(GSE20898)	-3,31E+03
FOXA1(Forkhead)	LNCAP-FOXA1-ChIP-Seq(GSE27824)	-3,28E+03
TRPS1(Zf)	MCF7-TRPS1-ChIP-Seq(GSE107013)	-3,12E+03
FOXK1(Forkhead)	HEK293-FOXK1-ChIP-Seq(GSE51673)	-3,12E+03
FOXA1(Forkhead)	MCF7-FOXA1-ChIP-Seq(GSE26831)	-3,06E+03
FoxL2(Forkhead)	Ovary-FoxL2-ChIP-Seq(GSE60858)	-3,05E+03
FOXK2(Forkhead)	U2OS-FOXK2-ChIP-Seq(E-MTAB-2204)	-2,93E+03
Gata6(Zf)	HUG1N-GATA6-ChIP-Seq(GSE51936)	-2,78E+03

F

Homer motif enrichment FOXA1
5637 specific peaks over SD48 specific peaks

Motif Name		Log P-value
Fra1(bZIP)	BT549-Fra1-ChIP-Seq(GSE46166)	-1,27E+03
Fos(bZIP)	TSC-Fos-ChIP-Seq(GSE110950)	-1,27E+03
Fra2(bZIP)	Striatum-Fra2-ChIP-Seq(GSE43429)	-1,23E+03
JunB(bZIP)	DendriticCells-Junb-ChIP-Seq(GSE36099)	-1,20E+03
Atf3(bZIP)	GBM-ATF3-ChIP-Seq(GSE33912)	-1,20E+03
BATF(bZIP)	Th17-BATF-ChIP-Seq(GSE39756)	-1,14E+03
AP-1(bZIP)	ThioMac-PU.1-ChIP-Seq(GSE21512)	-1,11E+03
Fosl2(bZIP)	3T3L1-Fosl2-ChIP-Seq(GSE56872)	-1,06E+03
Jun-AP1(bZIP)	K562-cJun-ChIP-Seq(GSE31477)	-9,24E+02

Figure S6: FOXA1 binding profile

A) MACS2 peakcalling in SD48 and 5637 statistics: Peak models for FOXA1 and FRiP (Fraction of Reads in Peaks). B) Examples washu genome browser view of Input, FOXA1 ChIPseq reads (raw bigwig as well as Fold enrichment compared to Input) and MAC2 called peaks at *GATA3* and *PPARg* genes. C) Genomic localisation of FOXA1 peaks. D) Cistrome ChIPseq enrichment analysis of 5637 and SD48 FOXA1 overlapping peaks. E) Homer motif enrichment analysis of FOXA1 SD48 specific FOXA1 peaks versus 5637 specific peaks. F) Homer motif enrichment analysis of FOXA1 5637 specific FOXA1 peaks versus SD48 specific peaks.
