# Supplementary material for: Epigenomic mapping identifies an enhancer repertoire that regulates cell identity in bladder cancer through distinct transcription factor networks
Source: Oncogene. 2023 Mar 22;42(19):1524–42. doi: 10.1038/s41388-023-02662-1 (PMC10162941; doi:10.1038/s41388-023-02662-1)
Supplement: Supplementary file 8 — Figure S7 [file 41388_2023_2662_MOESM8_ESM.pdf]

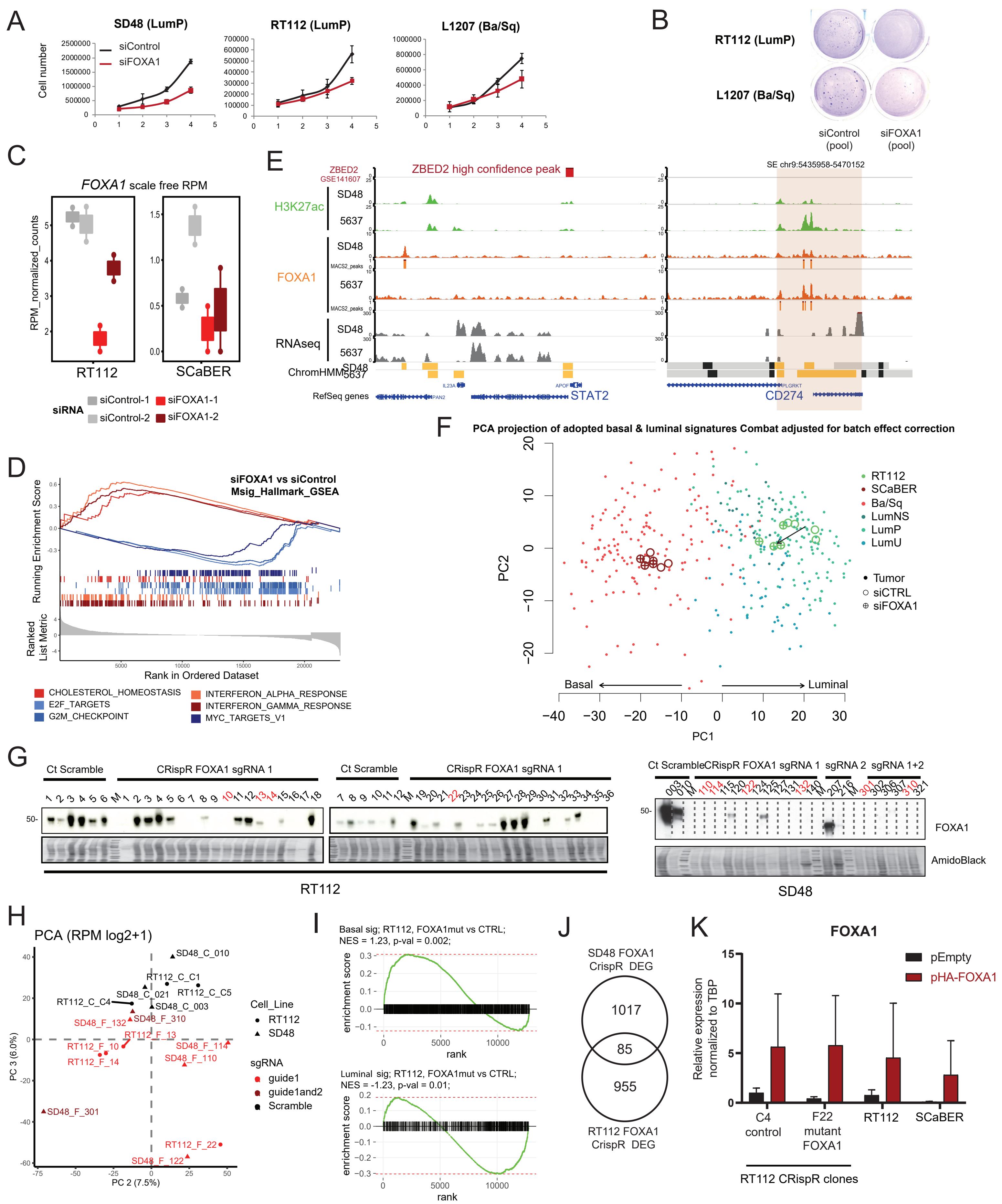

Figure S7: FOXA1 regulates inflammation and cellular identity

A) Proliferation assay of SD48, RT112 and L1207 cell lines after FOXA1 KD with a pool of siRNA. B) Soft agar assay of RT112 and L1207 upon FOXA1 KD. C) FOXA1 expression upon siRNA in RT112 and SCaBER cell lines. D) Msig Hallmark GSEA analysis of genes differentially regulated in siFOXA1 *vs* siControl samples (RT112 and SCaBER). E) Genome browser view of STAT2 and CD274 loci. H2K27ac and FOXA1 ChIPseq and RNA-seq in SD48 and 5637 cell lines. F) Projection of TCGA tumors, RT112 and SCaBER siFOXA1 samples on Luminal/Basal adopted signature. G) Western blot of SD48 and RT112 CRispR clones. Controls (Ct scramble), Mutants (FOXA1 sgRNA 1 or 2). H) PCA of CrispR clones 3'RNA-seq(Dim 2 and 3). I) GSEA analysis of RT112 FOXA1 mutant clones on Basal and Luminal signature. J) Venn diagram comparing differentially expressed genes in RT112 and SD48 FOXA1 CRispR clones. K) FOXA1 expression upon transfection of CRispR clones or RT112 and SCaBER by plasmid overexpressiong HA-FOXA1.
