# Supplementary material for: Epigenomic mapping identifies an enhancer repertoire that regulates cell identity in bladder cancer through distinct transcription factor networks
Source: Oncogene. 2023 Mar 22;42(19):1524–42. doi: 10.1038/s41388-023-02662-1 (PMC10162941; doi:10.1038/s41388-023-02662-1)
Supplement: Supplementary file 9 — Figure S8 [file 41388_2023_2662_MOESM9_ESM.pdf]

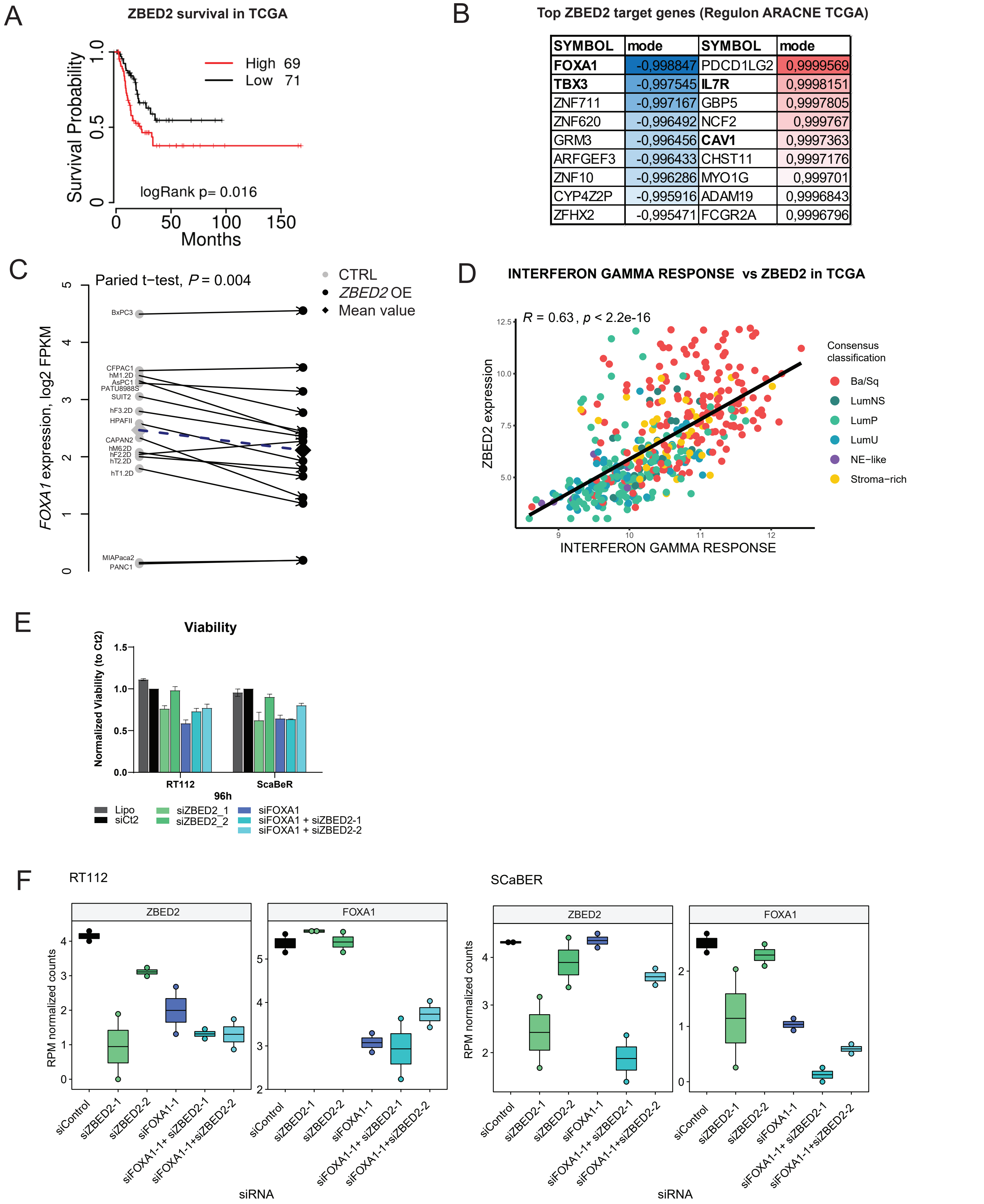

SYMBOL	mode	SYMBOL	mode
**FOXA1**	-0,998847	PDCD1LG2	0,9999569
**TBX3**	-0,997545	**IL7R**	0,9998151
ZNF711	-0,997167	GBP5	0,9997805
ZNF620	-0,996492	NCF2	0,999767
GRM3	-0,996456	**CAV1**	0,9997363
ARFGEF3	-0,996433	CHST11	0,9997176
ZNF10	-0,996286	MYO1G	0,999701
CYP4Z2P	-0,995916	ADAM19	0,9996843
ZFHX2	-0,995471	FCGR2A	0,9996796

Figure S8: ZBED2, a novel Basal-associated TF involved in inflammation dampening
A) Overall survival compared to ZBED2 expression in TCGA cohort (Mean +/- sd). B) Top ZBED2 target genes predicted by ARACNE from TCGA regulon. C) Statistical analysis of FOXA1 expression in 15 Pancreatic cell lines (GSE141607). D) Expression correlation of ZBED2 with Interferon Gamma response (Hallmark) in TCGA cohort. E) Viability assay of RT112 and SCaBER upon siRNA treatments (n=2). F) 3’seq analysis of RT112 and SCaBER after siRNA treatment against ZBED2 and FOXA1.
